# 1,25-dihydroxyvitamin D and PTHrP mediated malignant hypercalcemia in a seminoma

**DOI:** 10.1186/1472-6823-14-32

**Published:** 2014-04-10

**Authors:** René Rodríguez-Gutiérrez, Maria Azucena Zapata-Rivera, Dania Lizeth Quintanilla-Flores, Carlos Rodrigo Camara-Lemarroy, Fernando Javier Lavalle-Gonzalez, José Gerardo González-González, Jesús Zacarías Villarreal-Pérez

**Affiliations:** 1 Endocrinology Division, Internal Medicine Department, University Hospital “Dr. José E. González”, Ave. Madero y Ave. Gonzalitos s/n, Colonia Mitras Centro, Monterrey, Nuevo León 64460, Mexico; 2 Medical School of the Universidad Autónoma de Nuevo León, Ave. Madero y Ave. Gonzalitos s/n, Colonia Mitras Centro, Monterrey, Nuevo León 64460, Mexico; 3 Internal Medicine Department, University Hospital “Dr. José E. González”, Monterrey, Nuevo León 64460, Mexico

**Keywords:** 1,25-dihydroxyvitamin D, Calcitriol, PTHrP, Malignant hypercalcemia, Seminoma

## Abstract

**Background:**

Seminomas have been rarely associated with malignant hypercalcemia. The responsible mechanism of hypercalcemia in this setting has been described to be secondary to 1,25-dihydroxyvitamin D secretion. The relationship with PTHrP has not been determined or studied.

The aim of this study is to describe and discuss the case and the pathophysiological mechanisms involved in a malignant hypercalcemia mediated by 1,25-dihydroxyvitamin D and PTHrP cosecretion in a patient with seminoma.

**Case presentation:**

A 35-year-old man was consulted for assessment and management of severe hypercalcemia related to an abdominal mass. Nausea, polyuria, polydipsia, lethargy and confusion led him to the emergency department. An abdominal and pelvic enhanced CT confirmed a calcified pelvic mass, along with multiple retroperitoneal lymphadenopathy. Chest x-ray revealed “cannon ball” pulmonary metastases. The histopathology result was consistent with a seminoma. Serum calcium was 14.7 mg/dl, PTH was undetectable, 25-dihydroxyvitamin D was within normal values and PTHrP and 1,25-dihydroxyvitamin were elevated (35.0 pg/ml, and 212 pg/ml, respectively). After the first cycle of chemotherapy with bleomycin, etoposide and cisplatin, normocalcemia was restored. Both PTHrP and 1,25-dihydroxyvitamin D, dropped dramatically to 9.0 pg/ml and 8.0 pg/ml, respectively.

**Conclusion:**

The association of seminoma and malignant hypercalcemia is extremely rare. We describe a case of a patient with a seminoma and malignant hypercalcemia related to paraneoplastic cosecretion of 1,25-dihydroxyvitamin D and PTHrP. After successful chemotherapy, calcium, PTHrP and 1,25-Dihydroxyvitamin D returned to normal values.

## Background

Malignant hypercalcemia is the most common paraneoplastic syndrome occurring in 20-30% of patients with cancer [1]. It usually has an ominous prognosis, with a mortality of 50% within the next 30 days [2]. Both solid and hematologic malignancies have been associated with this syndrome; the most frequent etiologies being multiple myeloma, lung and breast cancer [3]. Four mechanisms of hypercalcemia in malignancy have been described: local osteolysis, parathyroid hormone related-protein (PTHrP) mediated, 1,25-dihydroxyvitamin D (1,25(OH)_2_D_3_, calcitriol) secretion, and ectopic parathyroid hormone (PTH) [4,5].

Seminomas have seldomly been related with hypercalcemia. Less than ten cases have documented this exceptional association. Besides two cases related to bone metastasis, the mechanism of hypercalcemia in seminomas has been discussed in two previous reported cases to be secondary to 1,25(OH)_2_D_3_ secretion. The underlying mechanism in the rest of the cases was not established. In the previous reports PTHrP was not measured nor related to hypercalcemia. In all of the previous documented cases, independent of the hypercalcemia related mechanism, normocalcemia was achieved soon after definitive treatment was initiated [6-9].

Herein, we present a case of a patient with a seminoma and malignant hypercalcemia mediated by 1,25(OH)_2_D_3_ and PTHrP paraneoplastic cosecretion.

## Case presentation

A 35-year-old man was referred to the Endocrinology Division for assessment and management of severe hypercalcemia related to an abdominal mass. Otherwise, he had an unremarkable medical history. One year before admission he noticed a painless hypogastric abdominal mass. The tumor progressively grew and two months before admission he developed asthenia, anorexia, general weakness, constipation, and an unintentional 10 kg weight loss. A month before admission the appearance of a left inguinal mass finally made him seek medical attention.

**Figure 1:**
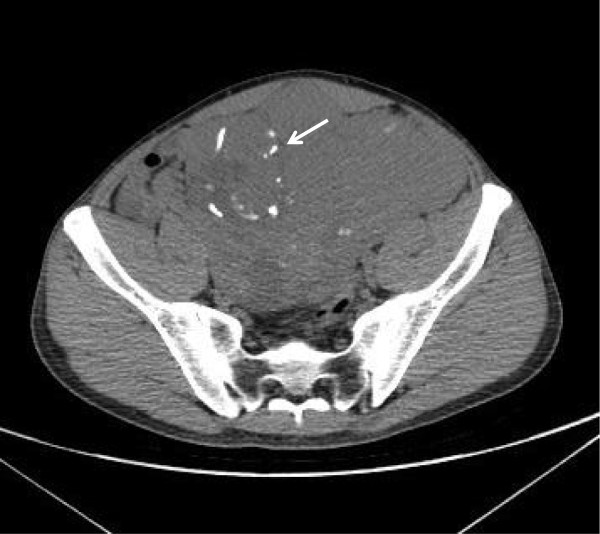
Pelvic enhanced computed tomography that shows a 10×9×7 cm mass with heterogeneous density and calcifications (Arrow).

An abdominal ultrasound revealed a 10 × 9 × 7 cm heterogeneous (hypoechogenic with hyperechogenic areas) retroperitoneal mass along with bilateral severe hydronephrosis. Serum creatinine was 2.8 mg/dl with a modification of diet in renal disease (MDRD) calculated glomerular filtration rate (GFR) of 35 ml/min. This consequently led to an uncomplicated unilateral left nephrostomy. An abdominal and pelvic enhanced computed tomography (CT) confirmed a calcified pelvic mass, along with multiple retroperitoneal lymphadenopathy (>5 cm each) (Figure 1). A biopsy was performed and the histopathology result was consistent with a seminoma. Chest x-ray revealed “cannon ball” pulmonary metastases. Lactate dehydrogenase (LDH) was 648 UI/L and human chorionic gonadotropin was slightly elevated (35.0 μUI/ml). Alpha-fetoprotein was within its normal reference value (Table 1). A stage IIIB seminoma was diagnosed and the patient was referred to oncology for chemotherapy.

**Table 1:** Laboratory measures

**Value**	**Basal**	**Post-chemotherapy**	**Range**
Glucose (mg/dl)	93	87	(70-100)
Creatinine (mg/dl)	3.7	2.8	(0.6-1.2)
Urea nitrogen (mg/dl)	11	13	(8-23)
MDRD GFR (ml/min)	24.0	37.1	(≥60)
Albumin (g/dl)	3.9		(3.5-5.0)
Calcium (mg/dl)	14.7	9.8	(8.2-10.2)
Phosphorus (pg/ml)	4.1	4.3	(2.3-4.7)
Magnesium (mg/dl)	1.8	2.1	(1.5-2.3)
Potassium (mmol/l)	3.8	4.1	(3.5-5.0)
Urinary calcium (mg/kg/d)	3.2	2.4	(2-4)
Alkaline phosphatase (UI/l)	146		(50-120)
LDH (UI/l)	648	110	(100-200)
AFP (ng/ml)	4.33	3.11	(≤6.0)
β-hCG (uUI/ml)	35.0		(≤3.0)
PTH (pg/ml)	<3.0	13.2	(1.5-37)
PTHrP (pg/ml)	35.0	9.0	(14-27)
25(OH)D_3_ (ng/ml)	22.0	21.0	(>20)
1,25(OH)_2_D_3_ (pg/ml)	212.0	8.0	(18-38)

MDRD GFR, modification of diet in renal disease glomerular filtration rate; LDH, lactate dehydrogenase; APF, alpha-fetoprotein; β –hCG, human chorionic gonadotropin; PTH, parathyroid hormone; PTHrP, parathyroid hormone related protein; 25(OH)D_3_, 25, Hydroxyvitamin D; 1,25(OH)_2_D_3_, 1,25-Dihydroxyvitamin D.

Two days before admission he presented nausea, polyuria, polydipsia, lethargy, and confusion that led him to the emergency room. On physical examination he was hemodynamically stable with a heart rate of 82 per minute, blood pressure of 130/85 mmHg, a respiratory rate of 16 per minute, a temperature of 36.3°C and a room-air oxygen saturation of 97%. An irregular 10 cm abdominal mass was palpated along with bilateral inguinal adenopathy. The left testicle was not palpable. Serum calcium was 14.7 mg/dl, albumin 3.9 g/dl, alkaline phosphatase 146 UI/l, creatinine 3.7 mg/dl with a MDRD GFR of 24 ml/min. He also had a mild elevated anion-gap metabolic acidosis and a urinary calcium of 3.2 mg/kg/d (240 mg/24 h). Phosphorous, magnesium, potassium, sodium, chlorine, and glucose were normal. PTH was undetectable, 25-dihydroxyvitamin D was normal, PTHrP was 35.0 pg/ml, and calcitriol 212 pg/ml (Quest Diagnostics Nichols, Chantilly, VA) (Table 1). An electrocardiogram showed a shortened QT interval and a bone scan was negative for metastasis. Calcitonin and hydration with intravenous saline solution at 250 ml/h was started. Despite therapy, calcium lowered only to 13.5 mg/dl, but nevertheless his mental status improved. The patient received four cycles of bleomycin, etoposide and carboplatin. After the first cycle of chemotherapy, calcium, LDH and alkaline phosphatase returned to normal values. Both PTHrP and 1,25(OH)_2_D_3_ dropped dramatically to 9.0 pg/ml and to 8.0 pg/ml, respectively (Table 1). At 8 months follow-up, the patient was asymptomatic. CT scans showed no residual disease and calcium levels have been consistently normal without medication.

## Discussion

This case illustrates, to our knowledge, the first reported case of malignant hypercalcemia in a patient with seminoma related to paraneoplastic cosecretion of PTHrP and 1,25(OH)_2_D_3_. Pure seminomas (no non-seminomatous elements present) account for up to 60% of all testicular germ cell tumors. They usually affect males between the second and fourth decade of life, representing 1% of all cancers in men. Undescended testes (cryptorchidism), as in our patient, is a risk factor and they are usually associated with a good prognosis with an overall five-year survival of 95% and even 80% in intermediate and high-risk patients [10,11]. Hyercalcemia associated with malignancy has been well described in both solid and hematologic malignancies (usually lymphomas) and represent the most common cause of elevated serum calcium in hospitalized patients [1]. The four mechanisms by which malignant hypercalcemia can occur are: 1) direct osteolytic metastases (20% of cases), usually related to multiple myeloma and breast cancer with release of cytokines such as interleukin (IL)-1, IL-6, IL-8 and activation of nuclear factor kappa beta; 2) PTH-rP or humoral hypercalcemia (80% of cases) usually described in nonmetastatic solid tumors (bladder, breast and squamous cell) and some non-Hodgkin lymphomas; 3) ectopic PTH secretion with only a few cases reported, and 4) 1,25(OH)_2_D_3_ secretion that accounts for less than 1% of cases [4,5,12,13].

Usually associated to granulomatous diseases such as sarcoidosis, tuberculosis, and systemic fungal infections, among others, calcitriol mediated hypercalcemia has been infrequently related to neoplasms [14]. Lymphomas have been the most common type of malignancy described, and other kind of tumors, such as disgerminomas, have rarely been reported [15]. Seminomas have been associated with malignant hypercalcemia almost anecdotally, with less than ten cases reported in the literature (Table 2). King et al. and Metcalfe et al. described the first two cases in the 70s. In their report, they both associated a seminoma with a pseudohyperparathyroidism and were not able to measure neither PTHrP nor vitamin D metabolites. In both cases, hypercalcemia resolved after definitive treatment with orchiectomy or corticosteroid-radiotherapy [6,7]. In 1987, Grote and Hainsworth reported the first case of a seminoma with a calcitriol mediated malignant hypercalcemia. Baseline calcitriol level was 125 pg/ml, with a normal PTH, 25-Hydroxyvitamine D and high 24 h urine calcium. PTHrP was not measured. After the first chemotherapy cycle, calcitriol concentration fell to 29 pg/mL and normocalcemia was achieved [8]. In 1992, da Silva et al. reported seven cases (four new and three previously described cases) of seminoma associated with malignant hypercalcemia. With regard to the four new cases, PTH was normal in three and was not measured in one. PTHrP and 25-hydroxyvitamin D were not measured in any case and 1,25(OH)_2_D_3_ was measured only in one case with an initial concentration of 79 pg/mL that decreased to 5 pg/mL after two cycles of chemotherapy and was associated afterwards with normocalcemia [9].

**Table 2:** Reported malignant hypercalcemia in seminomas

Author (*)	**Ca+**	**PTH**	**PTHrP**	**25D**	**1,25D**	**Tx**	**Ca + after Tx.**	**Outcome**
King [6]	15.4	NS	NS	NS	NS	Orchiectomy	NL	NED 10 mo.
Metcalfe [7]	15.8	NS	NS	NS	NS	GC + RT	NL	NED 8 mo.
Grote [8]	15.4	10.0	NS	24.0	112	BEP	NL	NED 2 yr.
Da Silva [9]	12.7	NL	NS	NS	NS	NSCT	NL	Adenophaty
Da Silva [9]	18.5	NS	NS	NS	NS	BEP	NL	NED 2 mo.
Da Silva [9]	20.1	NS	NS	NS	NS	NSCT	NL	NED 4 mo.
Da Silva [9]	16.4	0.3	NS	NS	79	BEP	NL	Improved after CT
This report	14.7	≤3.0	35.0	22.0	212	BEP	NL	NED 8 mo.

(*) Reference.

Ca+, Calcium (mg/dl); PTH, parathyroid hormone; PTHrP, parathyroid hormone related protein; 25D, 25, Hydroxyvitamin D; 1,25D, 1,25-Dihydroxyvitamin D; Tx, treatment; NS, not stated; NL, normal; NED, not evidence of disease; BEP, bleomycin, etoposide and cisplatinum; NSCT, not specified chemotherapy; CT, chemotherapy.

On the other hand, PTHrP has never been described in a patient with a pure seminoma. There are two reported cases of hypercalcemia associated with non-seminomatous germ cell tumors (NSGCTs). MacDiarmid et al. in 1995 reported a malignant hypercalcemia associated to an extragonadal NSGCT that was suspected to be probably due to a humoral hypercalcemia after bone scans were negative. PTHrP and vitamin D metabolites were not measured [16]. Later on, Sorscher described a patient with severe hypercalcemia and a NSGCT with a high PTHrP and a normal PTH that returned to normal values after chemotherapy. No measurements of 25-hydroxyvitamin D and 1,25(OH)_2_D_3_ levels were made [17]. In our case, a diagnosis of pure seminoma was well documented. Baseline serum calcium was elevated, urinary calcium, phosphorous and 25-hydroxyvitamin D were normal, PTH was undetectable and calcitriol and PTHrP were elevated. After one cycle of chemotherapy, normocalcemia was restored and both, calcitriol and PTHrP dropped to normal levels.

The exact mechanism by which calcitriol is produced in malignancy remains open to debate. Normally, in the kidney, 1α-hydroxylase converts 25-hydroxyvitamin D into the biologically active 1,25(OH)_2_D_3_, which is essential for bone and calcium metabolism/homeostasis. Nevertheless, 1α-hydroxylase has also been described in normal human tissues such as placenta, skin, gastrointestinal tract, testes, central nervous system and osteoblasts [18]. More than twenty granulomatous conditions are known to have enhanced 1α-hydroxylation of vitamin D in macrophages and increased production of active calcitriol causing hypercalcemia and/or hypercalciuria [19]. In lymphomas, Hewison et al., using inmunolocalization, showed stained sections of spleen that were negative for 1α-hydroxylase in lymphoma cells, but that were positive in surrounding macrophages [20]. Nonetheless, Evans et al. showed by reverse transcription polymerase chain reaction that mRNA for 1α-hydroxylase was increased 222-fold in disgerminoma cells compared to the surrounding non-ovarian tissue [21]. The exact mechanism in seminomas has not been explored. On the other hand, PTHrP is well recognized to be secreted by a variety of malignancies, in which there is an uncoupling of bone resorption and formation and a reduced ability of the kidney to clear calcium with the resultant hypercalcemia [22]. This last effect on the kidney could be the cause of the normal 24-h urinary calcium reported in our case, when it has been usually reported to be high in other calcitriol mediated pathologies [6-9,23]. As well, PTHrP is usually associated with hypophosphatemia due to an increase in urinary phosphorous; in this case, the concomitant renal failure could have diminished this last effect [24]. Interestingly, normocalcemia has consistently been achieved and sustained, in our case and in all previous cases, soon after definitive treatment with chemotherapy, surgery or glucocorticoids [5-8]. In this way, it is probable that bisphosphonates, denosumab, gallium nitrate, cinacalet and other medications directed to lower serum calcium could be withheld until definitive treatment is initiated.

## Conclusion

Seminomas have seldom been associated with hypercalcemia. We have described the case of a patient with a seminoma and malignant hypercalcemia related to cosecretion of 1,25(OH)_2_D_3_ and PTHrP. After successful treatment, calcium, PTHrP and 1,25(OH)_2_D_3_ returned to normal values. The exact mechanism by which seminomas secrete 1,25(OH)_2_D_3_ still remains to be elucidated.

### Consent

Written informed consent was obtained from the patient for publication of this case report and the accompanying images. A copy of the written consent is available upon request for review by the Journal Editor.

## Abbreviations

PTHrP: Parathyroid hormone related-protein; 1,25(OH)2D3: 1,25-dihydroxyvitamin D, calcitriol; PTH: Parathyroid hormone; MDRD GFR: Modification of diet in renal disease calculated glomerular filtration rate; LDH: Lactate dehydrogenase; IL: Interleukin; CT: Computed tomography; NSGCTs: Non-seminomatous germ cell tumors.

## Competing interests

The authors declare that they have no competing interests.

## Authors’ contributions

RRG and JGGG served as the principal investigators and led the conception, design, acquisition of data, review of literature, and drafted the manuscript. MAZR, DLQF, CRCL contributed in the acquisition of data, review of literature and reviewed the manuscript. FJLG and JZVP contributed the concept of research paper and critically reviewed the manuscript. All authors read and approved the manuscript.

## Authors’ information

RRG and MAZR are endocrinology fellows at the Endocrinology Division. DLQF and CRCL are internal medicine residents. FJLG is professor and chief of the diabetes clinic of the Endocrinology Division. JGGG is the vice-dean of research of the Medical School of the Universidad Autonoma de Nuevo Leon and professor of the Endocrinology Division. JZVP is the chief and director of the Endocrinology Division.

## Pre-publication history

The pre-publication history for this paper can be accessed here:

http://www.biomedcentral.com/1472-6823/14/32/prepub
